# Supplementary material for: Three New Species of Apiospora (Amphisphaeriales, Apiosporaceae) on Indocalamus longiauritus, Adinandra glischroloma and Machilus nanmu from Hainan and Fujian, China
Source: J Fungi (Basel). 2024 Jan 17;10(1):74. doi: 10.3390/jof10010074 (PMC10817522; doi:10.3390/jof10010074)
Supplement: Supplementary file 1 [file jof-10-00074-s001.zip › Table S1.pdf]

Table S1. Species and GenBank accession numbers of DNA sequences used in this study.

Species	Strain No.	Substrate	Country	GenBank Accession Numbers			
				ITS	LSU	TEF1α	TUB2
Apiospora acutiapica	KUMCC 20-0210 *	*Bambusa bambos*	China	MT946343	MT946339	MT947360	MT947366
A. adinandrae	**SAUCC 1282B-1 ***	***Adinandra glischroloma***	**China**	**OR739431**	**OR739572**	**OR753448**	**OR757128**
	**SAUCC 1282B-2**	***Adinandra glischroloma***	**China**	**OR739432**	**OR739573**	**OR753449**	**OR757129**
A. agari	KUC21333 *	*Agarum cribrosum*	Korea	MH498520	MH498440	MH544663	MH498478
A. aquatica	S-642 *	Submerged wood	China	MK828608	MK835806	–	–
A. arctoscopi	KUC21331 *	Egg of *Arctoscopus japonicus*	Korea	MH498529	MH498449	MN868918	MH498487
A. arundinis	CBS 124788	*Fagus sylvatica*	Switzerland	KF144885	KF144929	KF145017	KF144975
A. aurea	CBS 244.83 *	Air	Spain	AB220251	KF144935	KF145023	KF144981
A. balearica	CBS 145129 *	Undetermined *Poaceae*	Spain	MK014869	MK014836	MK017946	MK017975
A. bambusicola	MFLUCC20-0144 *	*Schizostachyum brachycladum*	Thailand	MW173030	MW173087	MW183262	–
A. bawanglingensis	**SAUCC BW0444 ***	***Indocalamus longiauritus***	**China**	**OR739429**	**OR739570**	**OR753446**	**OR757126**
	**SAUCC BW04441**	***Indocalamus longiauritus***	**China**	**OQ592551**	**OQ615280**	**OQ613324**	**OQ613302**
A. biserialis	CGMCC 3.20135 *	Bamboo	China	MW481708	MW478885	MW522938	MW522955
A. camelliae-sinensis	LC5007 *	*Camellia sinensis*	China	KY494704	KY494780	KY705103	KY705173
A. chiangraiense	MFLUCC21-0053 *	Dead culms of bamboo	Thailand	MZ542520	MZ542524	–	MZ546409
A. chromolaenae	MFLUCC 17-1505*	*Chromolaena odorata*	Thailand	MT214342	MT214436	–	–
A. cordylines	GUCC 10027 *	*Cordyline fruticosa*	China	MT040106	–	MT040127	MT040148
A. cyclobalanopsidis	CGMCC 3.20136 *	*Cyclobalanopsidis glauca*	China	MW481713	MW478892	MW522945	MW522962

A. descalsii	CBS 145130 *	*Ampelodesmos mauritanicus*	Spain	MK014870	MK014837	MK017947	MK017976
A. dichotomanthi	LC4950 *	*Dichotomanthus tristaniaecarpa*	China	KY494697	KY494773	KY705096	KY705167
A. dongyingensis	SAUCC 0302 *	Leaf of bamboo	China	OP563375	OP572424	OP573264	OP573270
	SAUCC 0303	Leaf of bamboo	China	OP563374	OP572423	OP573263	OP573269
A. esporlensis	CBS 145136 *	*Phyllostachys aurea*	Spain	MK014878	MK014845	MK017954	MK017983
A. euphorbiae	IMI 285638b	*Bambusa* sp.	Bangladesh	AB220241	AB220335	–	AB220288
A. fermenti	KUC21289 *	Seaweed	Korea	MF615226	MF615213	MH544667	MF615231
A. gaoyouensis	CFCC 52301 *	*Phragmites australis*	China	MH197124	–	MH236793	MH236789
A. garethjonesii	JHB004 *	Culms of dead bamboo	China	KY356086	KY356091	–	–
A. gelatinosa	HKAS 111962 *	Culms of dead bamboo	China	MW481706	MW478888	MW522941	MW522958
A. guiyangensis	HKAS 102403 *	Dead culms of *Poaceae*	China	MW240647	MW240577	MW759535	MW775604
A. guizhouensis	LC5322 *	Air in karst cave	China	KY494709	KY494785	KY705108	KY705178
A. hainanensis	SAUCC 1681 *	Leaf of bamboo	China	OP563373	OP572422	OP573262	OP573268
	SAUCC 1682	Leaf of bamboo	China	OP563372	OP572421	OP573261	OP573267
A. hispanica	IMI 326877 *	Maritime sand	Spain	AB220242	AB220336	–	AB220289
A. hydei	CBS 114990 *	Culms of *Bambusa tuldoides*	China	KF144890	KF144936	KF145024	KF144982
A. hyphopodii	MFLUCC 15-0003*	Dead culms of bamboo	Thailand	KR069110	–	–	–
A. hysterina	ICPM 6889 *	Bamboo	New Zealand	MK014874	MK014841	MK017951	MK017980
A. iberica	AP10118 *	*Arundo donax*	Portugal	MK014879	MK014846	MK017955	MK017984
A. piptatheri	**SAUCC BW0455 ***	***Indocalamus longiauritus***	**China**	**OR739430**	**OR739571**	**OR753447**	**OR757127**
	**SAUCC BW04551**	***Indocalamus longiauritus***	**China**	**OQ592550**	**OQ615279**	**OQ613323**	**OQ613301**
A. intestini	CBS 135835 *	Gut of grasshopper	India	KR011352	KR149063	KR011351	KR011350

A. italica	CBS 145138 *	*Arundo donax*	Italy	MK014880	MK014847	MK017956	MK017985
A. jatrophae	CBS 134262 *	*Jatropha podagrica*	India	JQ246355	–	–	–
A. jiangxiensis	LC4577 *	*Maesa* sp.	China	KY494693	KY494769	KY705092	KY705163
A. kogelbergensis	CBS 113333 *	Dead culms of *Restionaceae*	South Africa	KF144892	KF144938	KF145026	KF144984
A. koreana	KUC21332 *	Egg of *Arctoscopus japonicus*	Korea	MH498524	MH498444	MH544664	MH498482
A. locuta-pollinis	LC11683 *	*Brassica campestris*	China	MF939595	–	MF939616	MF939622
A. longistroma	MFLUCC 11-0481*	Culms of decaying bamboo	Thailand	KU940141	KU863129	–	–
A. malaysiana	CBS 102053 *	*Macaranga hullettii*	Malaysia	KF144896	KF144942	KF145030	KF144988
A. machili	**SAUCC 1175A-4**	***Machilus nanmu***	**China**	**OR739433**	**OR739574**	**OR753450**	**OR757130**
	**SAUCC 1175**	***Machilus nanmu***	**China**	**OQ592560**	**OQ615289**	**OQ613333**	**OQ613307**
A. marianiae	AP18219 *	Dead stems of *Phleum pratense*	Spain	ON692406	ON692422	ON677180	ON677186
A. marii	CBS 497.90 *	beach sands	Spain	MH873913	KF144947	KF145035	KF144993
A. marina	KUC21328 *	Seaweed	Korea	MH498538	MH498458	MH544669	MH498496
A. mediterranea	IMI 326875 *	Air	Spain	AB220243	AB220337	–	AB220290
A. minutispora	17E-042 *	Soil	South Korea	LC517882	–	LC518889	LC518888
A. montagnei	AP301120 *	*Arundo micrantha*	Spain	ON692408	ON692424	ON677182	ON677188
	AP19421	*Arundo micrantha*	Spain	ON692418	ON692425	ON677183	ON677189
	CPC 18900	Culms of *Phragmites australis*	Italy	KF144909	KF144956	KF145043	KF145001
A. mori	MFLU 18-2514 *	Dead leaves of *Morus australis*	China	MW114313	MW114393	–	–
A. multiloculata	MFLUCC 21-0023*	Dead culms of Bambusae	Thailand	OL873137	OL873138	–	OL874718
A. mytilomorpha	DAOM 214595 *	Dead blades of *Andropogon* sp.	India	KY494685	–	–	–
A. neobambusae	LC7106 *	Leaf of bamboo	China	KY494718	KY494794	KY806204	KY705186
A. neochinense	CFCC 53036 *	*Fargesia qinlingensis*	China	MK819291	–	MK818545	MK818547

A. neogarethjonesii	HKAS 102408 *	Dead culms of Bambusae	China	MK070897	MK070898	–	–
A. neosubglobosa	KUMCC 16-0203 *	Bamboo	China	KY356090	KY356095	–	–
A. obovata	LC4940 *	*Lithocarpus* sp.	China	KY494696	KY494772	KY705095	KY705166
A. ovata	CBS 115042 *	*Arundinaria hindsii*	China	KF144903	KF144950	KF145037	KF144995
A. paraphaeosperma	MFLUCC13-0644 *	Dead clumps of *Bambusa* sp.	Thailand	KX822128	KX822124	–	–
A. phyllostachydis	MFLUCC 18-1101*	*Phyllostachys heteroclada*	China	MK351842	MH368077	MK340918	MK291949
A. piptatheri	CBS 145149 *	*Piptatherum miliaceum*	Spain	MK014893	MK014860	MK017969	–
A. pseudomarii	GUCC 10228 *	Leaves of *Aristolochia debilis*	China	MT040124	–	MT040145	MT040166
A. pseudoparenchymatica	LC7234 *	Leaf of bamboo	China	KY494743	KY494819	KY705139	KY705211
A. pseudorasikravindrae	KUMCC 20-0208 *	*Bambusa dolichoclada*	China	MT946344	–	MT947361	MT947367
A. pseudosinensis	CPC 21546 *	Leaf of bamboo	Netherlands	KF144910	KF144957	KF145044	MN868936
A. pseudospegazzinii	CBS 102052 *	*Macaranga hullettii*	Malaysia	KF144911	KF144958	KF145045	KF145002
A. pterosperma	CPC 20193 *	Lepidosperma gladiatum	Australia	KF144913	KF144960	KF145046	KF145004
A. pusillisperma	KUC21321 *	Seaweed	Korea	MH498533	MH498453	MN868930	MH498491
A. qinlingensis	CFCC 52303 *	Fargesia qinlingensis	China	MH197120	–	MH236795	MH236791
A. rasikravindrae	LC5449	Soil in karst cave	China	KY494713	KY494789	KY705112	KY705182
A. sacchari	CBS 212.30	Phragmites australis	UK	KF144916	KF144962	KF145047	KF145005
A. saccharicola	CBS191.73	Air	Netherlands	KF144920	KF144966	KF145051	KF145009
A. sargassi	KUC21228 *	*Sargassum fulvellum*	Korea	KT207746	KT207696	MH544677	KT207644
A. sasae	CBS 146808 *	Dead culms of *Sasa veitchii*	Netherlands	MW883402	MW883797	MW890104	MW890120
A. septata	CGMCC 3.20134 *	Bamboo	China	MW481711	MW478890	MW522943	MW522960

A. serenensis	IMI 326869 *	Food, pharmaceutical excipients, atmosphere and home dust	Spain	AB220250	AB220344	–	AB220297
A. setariae	CFCC 54041 *	Decaying culms of *Setaria viridis*	China	MT492004	–	–	–
A.setostroma	KUMCC 19-0217	Dead branches of bamboo	China	MN528012	MN528011	MN527357	–
A. sichuanensis	HKAS 107008 *	Dead culms of *Poaceae*	China	MW240648	MW240578	MW759536	MW775605
A. sorghi	URM 93000 *	*Sorghum bicolor*	Brazil	MK371706	–	–	MK348526
A. sphaerosperma	CBS114314	Leaf of *Hordeum vulgare*	Iran	KF144904	KF144951	KF145038	KF144996
A. stipae	CBS 146804 *	Dead culm of *Stipa gigantea*	Spain	MW883403	MW883798	MW890082	MW890121
A.subglobosa	MFLUCC11-0397	Dead culms of bamboo	Thailand	KR069112	KR069113	–	–
A. subrosea	LC7292 *	Leaf of bamboo	China	KY494752	KY494828	KY705148	KY705220
A. taeanense	KUC 21322 *	Seaweed	South Korea	MH498515	–	MH544662	MH498473
A. thailandica	LC5630	Rotten wood	China	KY494714	KY494790	KY705113	KY806200
A. vietnamensis	IMI 99670 *	*Citrus sinensis*	Vietnam	KX986096	KX986111	–	KY019466
A. xenocordella	CBS 478.86 *	Soil from roadway	Zimbabwe	KF144925	KF144970	KF145055	KF145013
A. yunnana	MFLUCC 15-0002*	Decaying bamboo culms	China	KU940147	KU863135	–	–
Arthrinium caricicola	CBS 145127	*Carex ericetorum*	China	MK014871	MK014838	MK017948	MK017977

Notes: Ex-type strains are marked with "*", strains in this study are marked in bold and "–" .
