# Supplementary figures and images for: Three New Species of Apiospora (Amphisphaeriales, Apiosporaceae) on Indocalamus longiauritus, Adinandra glischroloma and Machilus nanmu from Hainan and Fujian, China
Source: J Fungi (Basel). 2024 Jan 17;10(1):74. doi: 10.3390/jof10010074 (PMC10817522; doi:10.3390/jof10010074)

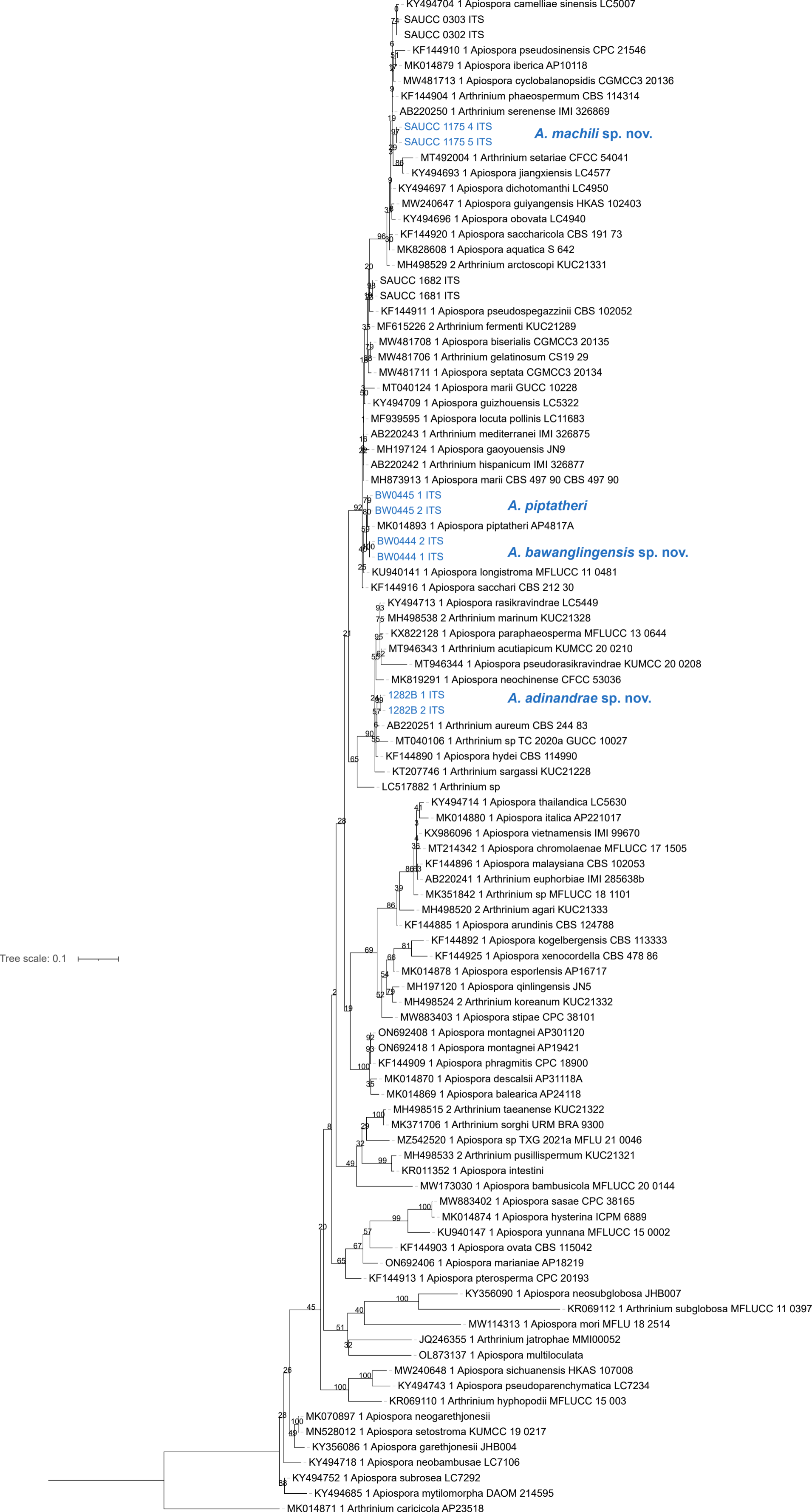

Supplement: Supplementary file 1 [file jof-10-00074-s001.zip › Figure S1. The ITS sequences Maximum Likelihood tree.pdf]

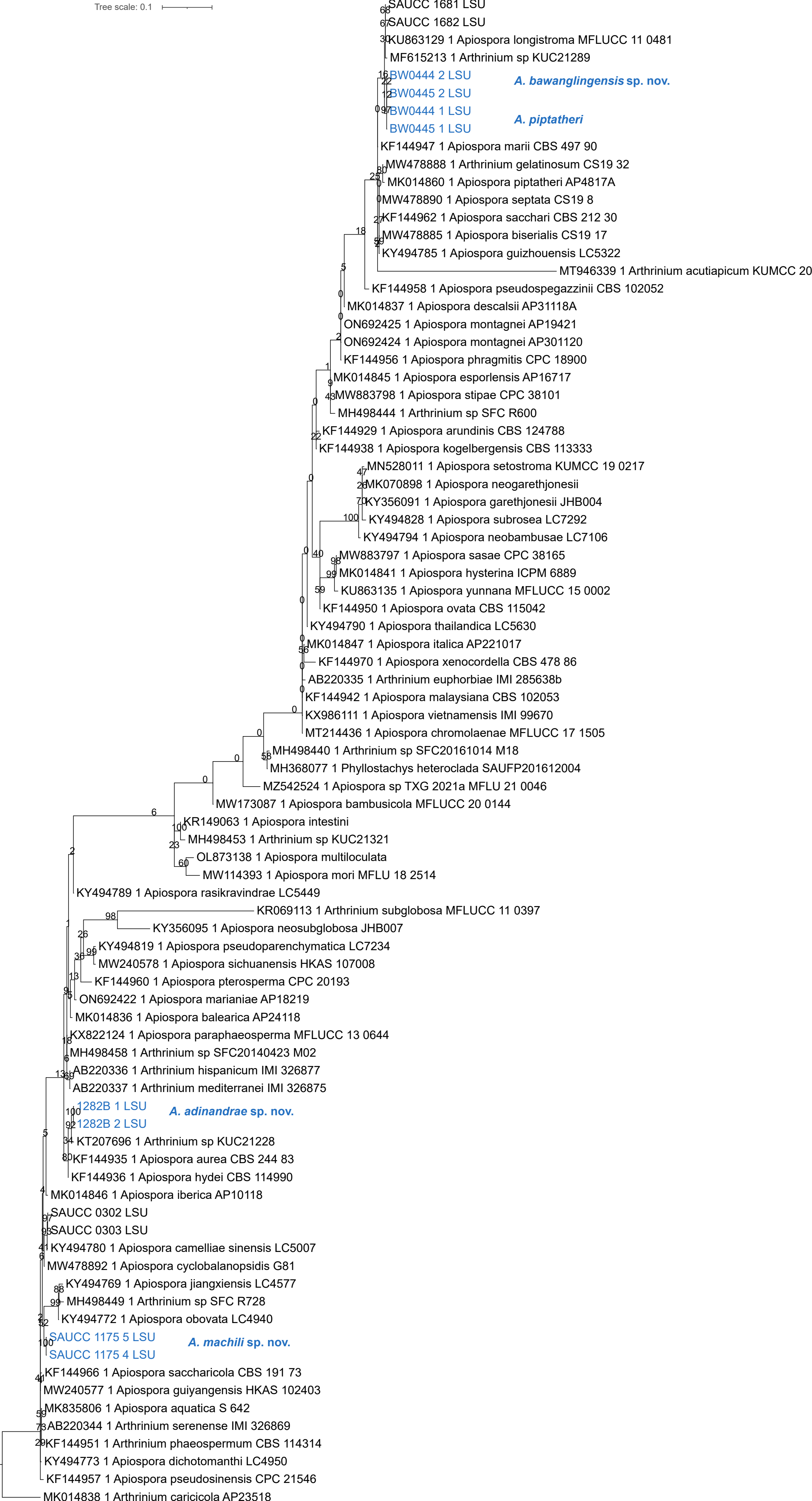

Supplement: Supplementary file 1 [file jof-10-00074-s001.zip › Figure S2. The LSU sequences Maximum Likelihood tree.pdf]

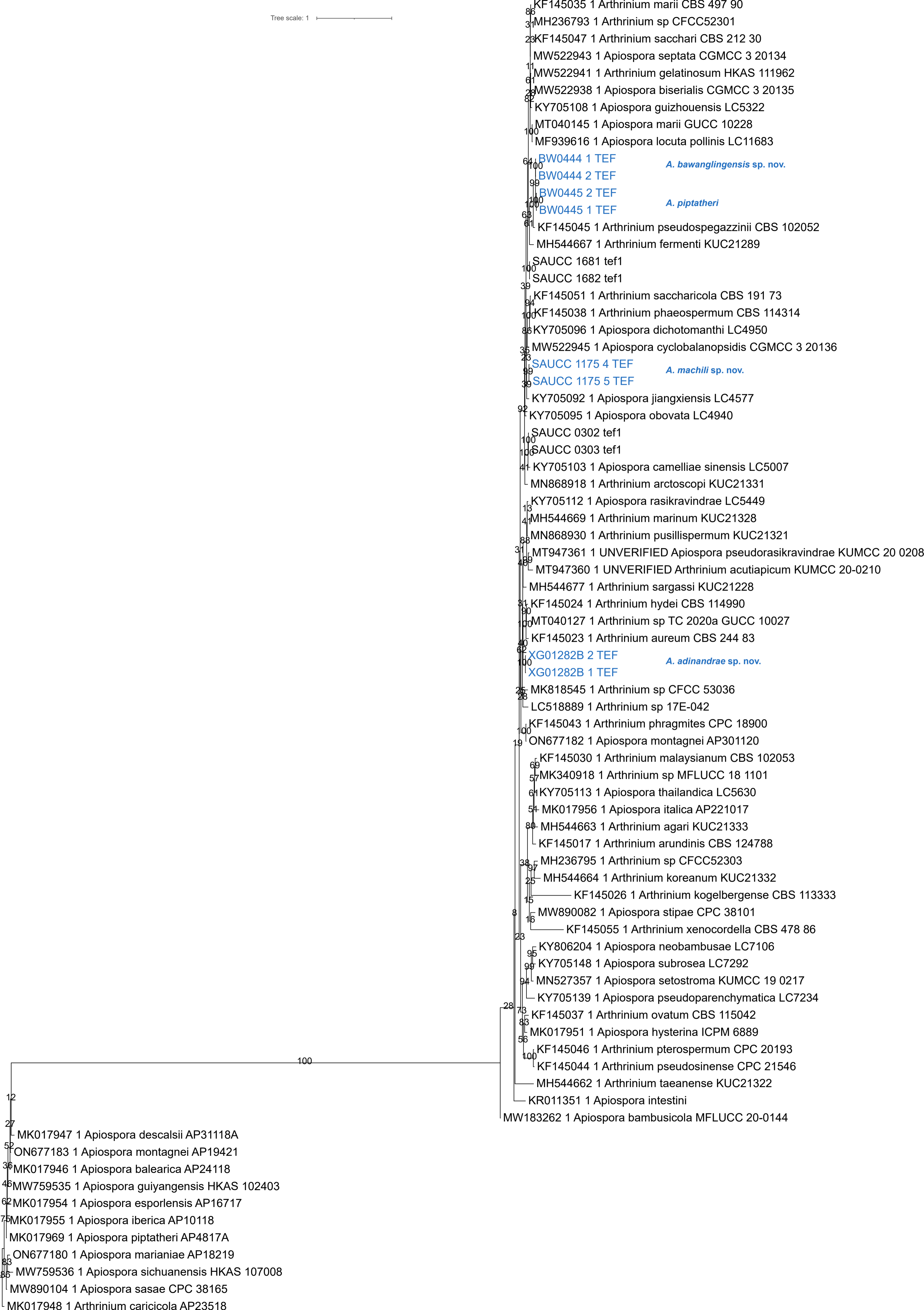

Supplement: Supplementary file 1 [file jof-10-00074-s001.zip › Figure S3. The TEF1a┴ sequences Maximum Likelihood tree.pdf]

Tree scale: 0.1

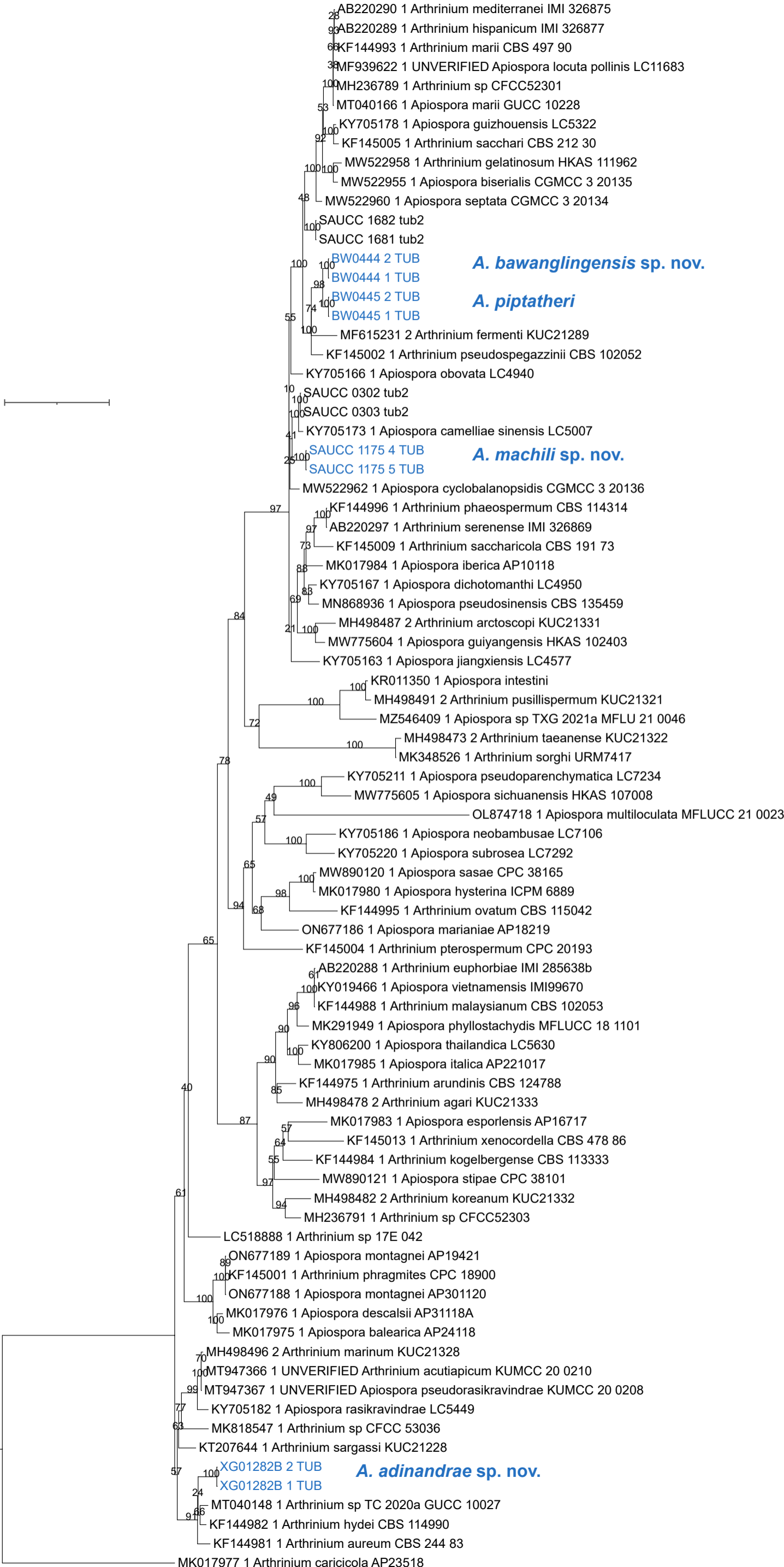

Supplement: Supplementary file 1 [file jof-10-00074-s001.zip › Figure S4.The TUB2 sequences Maximum Likelihood tree.pdf]
